# Methylated Spirit Drinking

**Published:** 1902-09-06

**Authors:** 


### METHYLATED SPIRIT DRINKING.

The methylated spirit of commerce is a compound containing 10 per cent. of wood naphtha, 2 per cent. of mineral oil, and 64 per cent. of absolute alcohol. It contains twice as much alcohol as the finest whisky and can be retailed at a considerable profit for 5d. per pint. There is no restriction as to its sale, provided that its composition conforms to the above formula, and that the retailer obtains a license which costs but 10s. a year, although somewhat recently the Inland Revenue Authorities have forbidden the sale of methylated spirits to the public between the hours of 10 o'clock on Saturday night and 8 o'clock on Monday morning. Dr. J. C. McWalter[1] clearly shows that illimitable possibilities for evil must result from this unrestricted sale of methylated spirit. There is no doubt but that large numbers seek in this agent a cheap intoxicant. Habitual drinkers have not been slow to discover that the oily constituents may at least in part be removed by dilution of the spirit with water and subsequently skimming. Both the wood naphtha and the mineral oil possess important toxic properties. We have in fact in the methylated spirit of commerce a fluid compounded of three powerful intoxicants, capable of producing intensely greater effects than the most virulent form of raw whisky. Dr. McWalter thinks the addition of naphthaline may prove an effectual deterrent. He also shows that the drinking of ether in the North of Ireland was stopped by the simple expedient of putting it on the schedule of poisons; and suggests that a like step taken in regard to methylated spirit would ensure its sale being confined to chemists and secure the labelling of it as "poison." Certainly measures should be speedily found which, while providing for the legitimate use of methylated spirit in the arts and as a convenient domestic fuel should prevent its use as "a beverage."

[1] The Journal of State Medicine, August 1902.

